# Unpacking the Experiences of Health Care Professionals About the Web-Based Building Resilience At Work Program During the COVID-19 Pandemic: Framework Analysis

**DOI:** 10.2196/49551

**Published:** 2024-01-31

**Authors:** Wei How Darryl Ang, Zhi Qi Grace Lim, Siew Tiang Lau, Jie Dong, Ying Lau

**Affiliations:** 1 Alice Lee Centre for Nursing Studies, Yong Loo Lin School of Medicine, National University of Singapore Singapore Singapore; 2 The Nethersole School of Nursing, Faculty of Medicine, The Chinese University of Hong Kong Hong Kong China (Hong Kong)

**Keywords:** resilience, intent to stay, employability, health care professionals, process evaluation, framework analysis, framework, resilience, stress, mental health disorder, prevention, training, qualitative study, web-based tool, tool, sustainability

## Abstract

**Background:**

The COVID-19 pandemic has resulted in a greater workload in the health care system. Therefore, health care professionals (HCPs) continue to experience high levels of stress, resulting in mental health disorders. From a preventive perspective, building resilience has been associated with reduced stress and mental health disorders and promotes HCPs’ intent to stay. Despite the benefits of resilience training, few studies provided an in-depth understanding of the contextual factors, implementation, and mechanisms of impact that influences the sustainability of resilience programs. Therefore, examining target users’ experiences of the resilience program is important. This will provide meaningful information to refine and improve future resilience programs.

**Objective:**

This qualitative study aims to explore HCPs’ experiences of participating in the web-based Building Resilience At Work (BRAW) program. In particular, this study aims to explore the contextual and implementational factors that would influence participants’ interaction and outcome from the program.

**Methods:**

A descriptive qualitative approach using individual semistructured Zoom interviews was conducted with participants of the web-based resilience program. A framework analysis was conducted, and it is guided by the process evaluation framework.

**Results:**

A total of 33 HCPs participated in this qualitative study. Three themes depicting participants’ experiences, interactions, and impacts from the BRAW program were elucidated from the framework analysis: learning from web-based tools, interacting with the BRAW program, and promoting participants’ workforce readiness.

**Conclusions:**

Findings show that a web-based asynchronous and self-paced resilience program is an acceptable and feasible approach for HCPs. The program also led to encouraging findings on participants’ resilience, intent to stay, and employability. However, continued refinements in the components of the web-based resilience program should be carried out to ensure the sustainability of this intervention.

**Trial Registration:**

ClinicalTrials.gov NCT05130879; https://clinicaltrials.gov/ct2/show/NCT05130879

## Introduction

### Background

The emergence of the COVID-19 pandemic has led to extensive changes in the health care landscape. Globally, the repeated waves of COVID-19 infections have led to health care professionals (HCPs) grappling with occupational health hazards and overstretched assignments [1,2]. These constant stressors have led to HCPs experiencing a surge in symptoms of burnout, insomnia, and mental health distress [3-5]. Accordingly, the intensification of physical and mental exhaustion has led to a considerable increase in the turnover of HCPs [6]. With a smaller health care workforce, health care administrators need to prioritize and concentrate their efforts on enforcing supportive measures to ensure that HCPs continue to be inoculated against stress and mental health disorders. Thus, reducing workplace-related stress may have encouraging effects on HCPs’ intent to stay [7,8].

Contemporarily, more persuasive evidence has alluded to the importance of noncognitive skills as protective factors against mental health distress [9,10]. An emerging interest among noncognitive skills is the development of an individual’s resilience. Resilience is the ability to overcome adversities [11,12]. Theoretically, resilience can be understood from various perspectives, as a trait (eg, personality), process (eg, interaction with protective factors), or outcome (eg, becoming resilient). More importantly, building an individual’s resilience has positive effects on their mental well-being [13,14].

Resilient individuals are adept at using personal, relational, and environmental resources to overcome adversity [11,12]. At the personal level, individuals with certain personality traits such as a positive outlook can appraise stressful situations from an optimistic point of view [15]. Based on the transactional model of stress and coping [16], positive emotions may reduce the negative effect that arises when one experiences adversities. Furthermore, individuals with collegial relationships with colleagues and peers can rely on social support resources to overcome adversities [11]. Finally, environmental protective factors in the form of workplace culture can influence an individual’s resilience [11,12]. For instance, an organization that focuses on building a collegial and harmonious workplace culture can in turn facilitate one’s access to social support resources and thus develop resilience [17,18].

**Figure 1:**
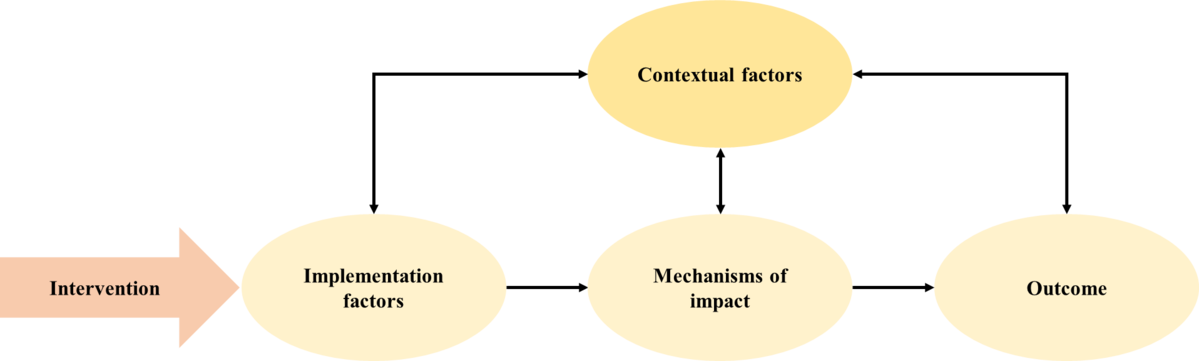
Process evaluation framework.

Existing resilience interventions have focused on modifiable personal and relational factors such as the use of cognitive behavioral techniques [19], mindfulness training [20,21], and social competency skills [22,23]. However, most existing literature focused on evaluating the effects of resilience training using quantitative approaches [13]. In line with the development and evaluation of complex interventions [24], using qualitative approaches will be useful in gathering in-depth information about the various contextual and implementational factors that can alter the intended outcomes of the intervention. Particularly, the process evaluation framework [25] proposes that an intervention should be further examined by identifying the contextual factors, implementation processes, mechanisms of impact, and outcomes of the intervention (Figure 1).

First, contextual factors are unique situational factors that influence how the intervention may be delivered or have affected the participants [25]. These contextual factors may have eventual implications on the implementation and mechanisms of impact. Second, the implementation process is the identification of factors that may influence the delivery of the intervention [25]. This may include the collection of data that reflects intervention fidelity [26]. Third, mechanisms of impact describe participants’ responses to and interaction with the intervention. In addition, mechanisms of impact identify any potential mediators, pathways, or consequences as a result of their participation in the intervention [25]. Thus, conducting process evaluations of interventions may be worthy in providing recommendations for improvements and supporting the eventual implementation of the program. Although prior qualitative evaluations of resilience programs [22,27,28] have made valuable contributions toward an in-depth understanding of participants’ experiences, its findings may not be transferrable because of several factors, such as population, cultural differences, and type of resilience program. For these reasons, conducting a study to encapsulate the experiences of the participants of the Building Resilience At Work (BRAW) program is important.

### Objectives

This qualitative study explores HCPs’ experiences of participating in the BRAW program. Guided by the process evaluation framework [25], this study also aims to examine the contextual and implementation factors that affected participants’ experiences and identify the outcomes that arose from their participation in the BRAW program.

## Methods

### Ethical Considerations

This study was approved by the National University of Singapore Institutional Review Board (NUS-IRB-2021-703). This study’s procedures were followed in accordance with the Declaration of Helsinki. Eligible participants were recruited from August 2021 to December 2022. Participants were provided with a participation information sheet, and they were allowed to withdraw without penalty. After obtaining informed consent, participants were invited to participate in a web-based semistructured audio- and video-recorded interview via Zoom (Zoom Video Communications). The interview transcripts were de-identified and coded using pseudonyms. Participants were given 20 Singapore Dollars for completing the study.

### Research Design

This qualitative study is part of a randomized controlled study conducted in Singapore (ClinicalTrials.gov NCT05130879). A process evaluation approach [25] comprising semistructured individual digital interviews was undertaken to explore participants’ experiences of using the web-based BRAW program. This study is reported based on the COREQ (Consolidated Criteria for Reporting Qualitative Research) [29] (Multimedia Appendix 1).

### Setting and Participants

This study was conducted from April 2021 to December 2022 in Singapore, a multiethnic and multicultural city-state. Based on the national census [30], there are approximately 70,178 registered HCPs, and most of them are nurses (61.27%). Participants were eligible to participate in this qualitative study if they were practicing as an HCP in Singapore, could comprehend the English language, had access to a device that could connect to the internet, and completed the web-based BRAW program. A total of 33 participants who completed the web-based BRAW program were purposively sampled to participate in this qualitative study.

### Web-Based BRAW Program

**Figure 2:**
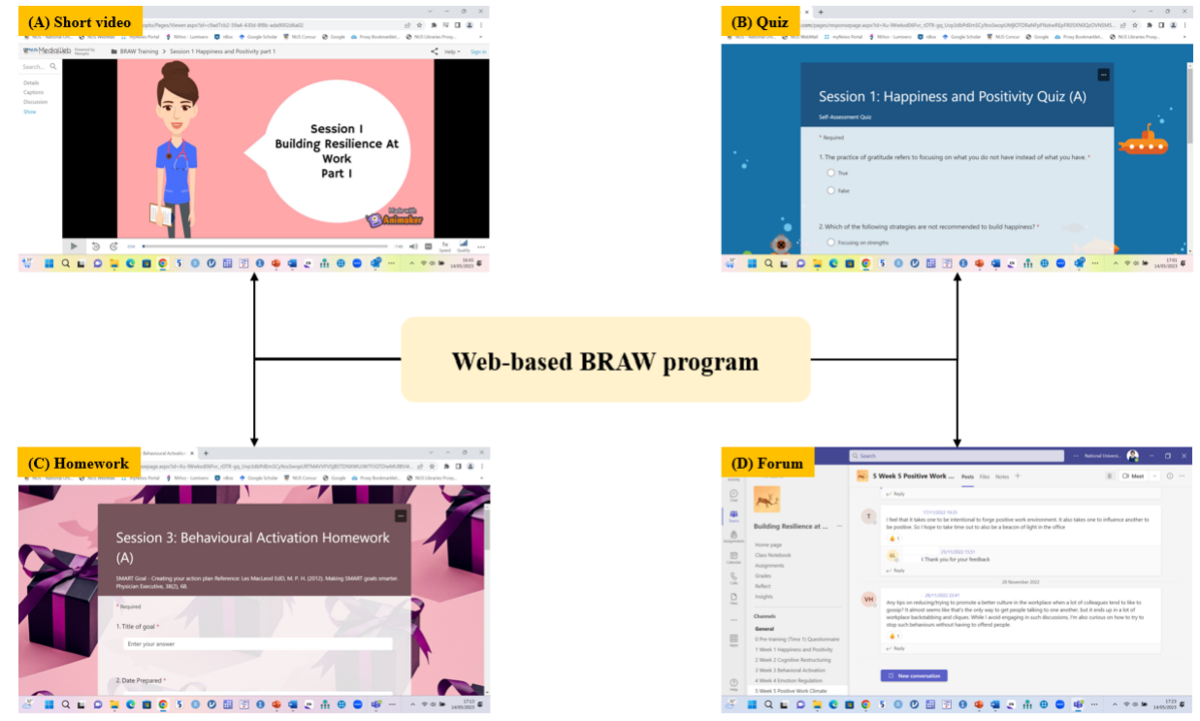
Elements of the web-based BRAW program. BRAW: Building Resilience At Work.

The web-based BRAW program is a 6-session weekly web-based program hosted via Microsoft Teams (Microsoft Corp). The resilience program was developed based on a systematic review [13] and evidence-based therapies, such as cognitive behavioral therapy [31], acceptance and commitment therapy [32], and problem-solving model [33]. The BRAW program comprised 6 different topics, namely, happiness and positivity, cognitive restructuring, behavioral activation, emotion regulation, positive work climate, and problem-solving (Table 1). It also comprised several elements, short videos, quizzes, and homework (Figure 2). A web-based forum was also provided for participants to interact with each other and provide social support.

**Table 1:** Overview of the Building Resilience At Work program.

Week	Topic	Contents
1	Happiness and positivity	Understanding strengths and resilience Fostering positive attitude
2	Cognitive restructuring	Identifying dysfunctional automatic thoughts Using cognitive behavioral techniques to modify dysfunctional thoughts Formulating rational responses to automatic thoughts
3	Behavioral activation	Initiating and using behavioral activation techniques Building healthy interpersonal relationships and peer support
4	Emotion regulation	Regulating emotions Preventing and managing conflict
5	Positive work climate	Forging a supportive work environment Developing supportive collegial relationships Promoting coworker support
6	Problem-solving	Solving work-life problems using a framework Importance of work-life balance

### Data Collection

The digital interviews were scheduled at a time convenient for the participants. Participants were reminded to ensure that their cameras and microphones were working prior to the interviews. All interviews were conducted by a female researcher (ZQGL) who received formal training in qualitative research. The interviewer was supported by 2 doctoral-prepared researchers (WHDA and YL) who are experienced in qualitative research. During the digital interview, the interviewer started by building rapport with the participants and sharing the aims and processes of this study. In addition, sociodemographic characteristics including age, sex, ethnicity, and occupation were collected. Afterward, the interview was conducted according to the semistructured guide. The guide was developed based on the process evaluation framework [25] and comprised open-ended questions. Then, the initial guide was circulated to the research team and refined. Subsequently, the interview guide was piloted among 5 participants and was further revised for clarity. The final interview guide can be found in Textbox 1. The mean duration of the interviews was 35.48 (SD 7.83; range 20-54) minutes. Data saturation was achieved at the 31st participant, and 2 additional interviews were conducted to confirm saturation [34].

**Semistructured interview guide.**

**Questions**

1. What was your experience when completing the Building Resilience At Work (BRAW) training program?
2. What were the issues with the platforms for the training sessions that you have encountered?
3. How did you feel about the duration of each training video?
4. How did you feel about the quizzes?
5. How did you feel about the homework?
6. How did you feel about the forum?
7. How did you feel about the entire duration of the 6-week BRAW training program?
8. What were the aspects of the intervention (eg, homework, quizzes, and forum) that you particularly liked or disliked?
9. Were there any sessions that stood out?
10. How did you feel about the contents?
11. Could you tell me your overall experience with applying the strategies learned from the BRAW intervention at work?
12. How was your experience of applying the strategies at work?
13. Did you encounter any problems or frustrations when trying to apply the strategies at work?
14. Has the BRAW training program influenced your resilience at work?
15. Has the BRAW training program influenced your enthusiasm and dedication at work?
16. Has the BRAW training program influenced your intention to leave?
17. Has the BRAW training program influenced your ability to gain and maintain employment?
18. Has the BRAW training program influenced your work performance?
19. Are there any other strategies that would help you to manage stress and build resilience that we have not mentioned in the BRAW intervention?
20. Do you have anything else to add that we have not covered in this interview?
21. Finally, are you okay for me to contact you for some follow-up questions?

### Data Analysis

The video-recorded interviews were transcribed verbatim by 1 researcher (ZQGL) and verified for accuracy by another researcher (WHDA). The transcripts were imported and analyzed using NVivo (version 12; Lumivero). Transcripts were returned to the participants for their comments. A deductive framework analysis method [35] was then undertaken as it provides a systematic approach to analyzing qualitative data [36]. In addition, the use of a matrix structure provides a visually straightforward recognition of patterns in the data that can be useful in identifying similarities or differences between participants’ narratives [36]. In line with the research questions, a framework analysis approach is suitable, as this study was guided by the process evaluation framework and sought to examine participants’ experiences of the BRAW program. Particularly, it identifies the contextual and implementation factors that affected their participation and the outcomes of participation.

A 5-step framework analysis approach [35,37] was independently performed by 2 researchers (WHDA and YL). First, the researchers familiarized themselves with the data by reading the transcripts accompanied by listening to the interviews. Second, the transcripts were coded based on the process evaluation framework [25]. After completing the coding for the first 5 transcripts, both researchers compared their codes and developed a standardized code book. Following discussions among the researchers, the eventual code book comprised 11 different categories.

Third, after completing the coding for all transcripts, a total of 347 codes were brought together and discussed among the researchers. The similarities and differences that arose during the coding process were deliberated. Cohen κ was used to calculate the interrater agreement for the coding, and good agreement was found (κ=0.79). Consequently, the codes were organized and indexed based on the process evaluation framework. Fourth, the codes were further reduced by summarizing the key information for the indexed data in each category. Finally, the identified codes were mapped using a coding tree (Table S1 Multimedia Appendix 2) and interpreted using visual and narrative forms. Finally, 3 themes and 7 subthemes were derived from the framework analysis. The themes and subthemes were provided to a select group of participants who were willing to provide feedback on the findings.

### Rigor

The principles of credibility, transferability, dependability, and conformability were used to demonstrate rigor [38]. First, a reflexivity journal was maintained by all members of the research team to improve their self-awareness and reduce any potential personal influences on the data. Second, the data analyses were conducted by 2 independent researchers (WHDA and YL). Third, participants were invited to review their transcripts to clarify the context of the statements and ensure that the final themes and subthemes were representative of their experiences [39]. Subsequently, an audit trail detailing the recruitment, data collection, and analysis process was conducted to ensure ease of replication, transparency, and dependability [38]. Finally, a thick description of the context and the intervention was provided, this facilitates the transferability of the findings of this study [38].

## Results

### Overview

**Figure 3:**
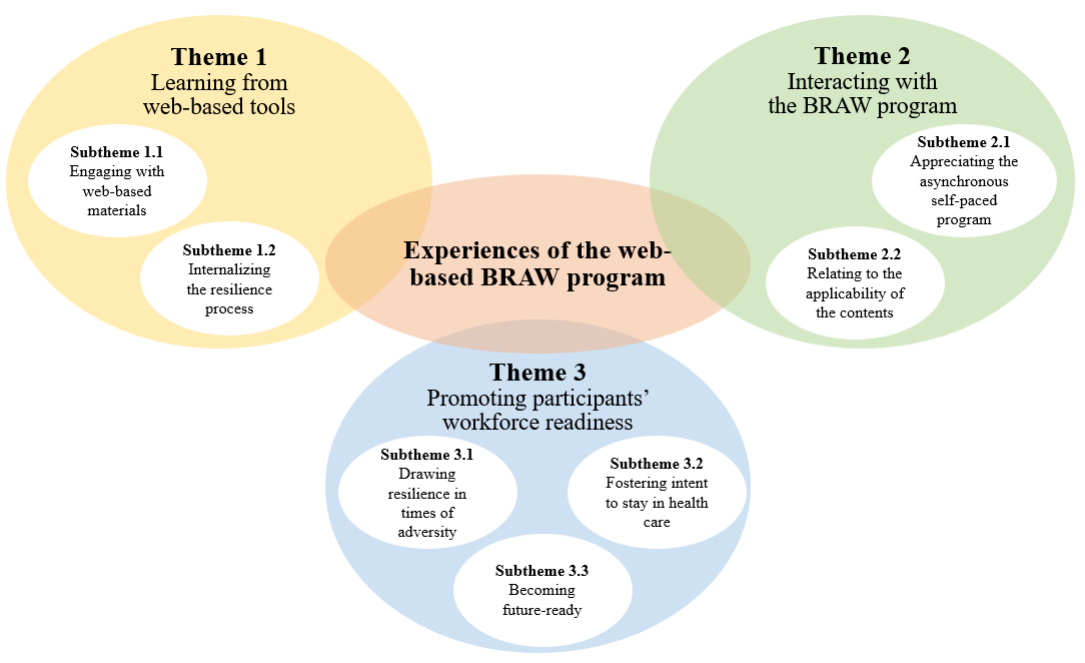
Participants’ experiences of the web-based BRAW program. BRAW: Building Resilience At Work.

A total of 33 HCPs participated in this qualitative study. The sociodemographic variables are presented in Table 2. Most of the participants were between the ages of 31-40 years (n=11, 34%), female (n=24, 73%), ethnic Chinese (n=25, 76%), and nurses (n=15, 46%). The findings from the framework analysis unveiled 3 themes and 7 subthemes that depicted participants’ experiences, interactions, and impacts from the BRAW program. The 3 themes were learning from web-based tools, interacting with the BRAW program, and promoting participants’ workforce readiness (Figure 3).

**Table 2:** Participants sociodemographic characteristics (N=33).

Variables		Values
**Age group (years), n (%)**		
	21-25	5 (15)
	26-30	9 (27)
	31-40	11 (34)
	41-50	6 (18)
	51-60	2 (6)
**Sex, n (%)**		
	Male	9 (27)
	Female	24 (73)
**Ethnicity, n (%)**		
	Chinese	25 (76)
	Malay	7 (21)
	Indian	1 (3)
**Profession, n (%)**		
	Allied health worker	12 (36)
	Clinical administrator	1 (3)
	Clinical researcher	4 (12)
	Nurse (registered and enrolled)	15 (46)
	Physician	1 (3)
**Duration of interviews (minutes)**		
	Mean (SD)	35.48 (7.83)
	Range	20-54

### Theme 1: Learning From Web-Based Tools

#### Overview

The first theme depicts the BRAW implementation process. It particularly describes how participants learned through web-based tools via Microsoft Teams. This is elaborated in 2 subthemes, namely, engaging with web materials and internalizing the resilience process.

#### Engaging With Web Materials

The BRAW program provided various web materials, ranging from short videos to quizzes and homework. The short videos were developed using animations, graphics, and subtitles, which appealed to the participants and supported their engagement with the web materials:

The use of graphics was quite good, the animations and all, so like, it kept me wanting to finish watching, not like stop halfway. Yeah...the pace was also good, and like, just nice, not too much information overload.

Participant 24, female, Chinese, nurse

However, some participants were encumbered by the number of tasks (eg, weekly quizzes and homework). For instance, the weekly homework was described to be a “chore,” and this can be a disincentivizing factor in completing the program. As an alternative, a participant proposed that renaming the weekly tasks could be a strategy to overcome the inertia:

Because “homework” it sounds like “tsk,” erm, like a chore to be done, you know, but “reflection” is like, you reflect on what you-you-you need to do. So, sounds more forgiving.

Participant 26, female, Malay, nurse

#### Internalizing the Resilience Process

Despite the conflicting work commitments and activities in the BRAW program that participants had to undergo, they credited the quizzes and homework as factors that supported the internalization of the learning process. Particularly, reviewing the questions found in the quizzes and homework facilitated an internalization process:

Just by plain reading the question, it may set you thinking, you see. You don’t know what’s happening or your subconscious, you’re already motivated right, you learn some new content. And that homework may actually be building synapses, you know, trying at the backend that you don’t know about.

Participant 10, male, Malay, physician

However, not all participants were well-versed in the contents of the BRAW program. Several participants highlighted difficulties in appreciating the theoretical aspects of the program:

When it gets a little bit more “science-y,” like the brain and then they tell you, I don’t know all the words, I don’t remember, but like the brain and then, certain kinds of thoughts and all that. Then, those kinds of stuff, no, like I haven’t heard of that before.

Participant 15, female, Indian, clinical researcher

Notwithstanding, these groups of participants, particularly those who did not receive formal training in health sciences, verbalized how they used the quizzes as an avenue to understand the various technical terms that they were not familiar with:

Especially some of the terms, erm, maybe a bit technical? I’m not that acquainted. So, it [referring to the quizzes] allows me to clarify, review and understand and get it correct.

Participant 8, female, Chinese, clinical administrator

### Theme 2: Interacting With the BRAW Program

#### Overview

The second theme describes the BRAW program’s mechanism of impact and the relevant contextual factors that influenced it. This theme expressed how participants responded and interacted with the BRAW program and is highlighted in 2 subthemes, namely, appreciating the asynchronous self-paced program and relating to the applicability of the contents.

#### Appreciating the Asynchronous Self-Paced Program

Due to the higher workload brought upon by the COVID-19 pandemic and the resumption of usual clinical duties, participants had to contend with numerous conflicting priorities. Hence, they appreciated how the BRAW program was designed as an asynchronous self-paced program. This allowed them to learn at their own pace and time:

Healthcare workers are busy, so they don’t have to find a specific day and time to attend an intervention, whether be it online or on-site, face-to-face or whatever, so having something that you can access on your own time and target is good.

Participant 4, female, Chinese, clinical researcher

However, despite the self-paced nature of the program, participants struggled with finding suitable time outside their personal commitments and rest to engage in the program. This was more prominent among HCPs who are on shift work duties:

We are really packed and rushed at work, and there’s a lot of multitasking. It’s like very draining at work. I think the shifts also, so you do rotating shifts. So, it’s quite tiring after work to find time.

Participant 5, female, Chinese, nurse

Nevertheless, some participants felt that introducing more web-based synchronous elements through videoconferencing tools may be able to better support their learning:

These sessions were to be interactive whereby we can do it via Zoom, to share every participant’s experience, it would be even better.

Participant 28, female, Chinese, nurse

#### Relating to the Applicability of the Contents

The BRAW program was conducted at the peak of the COVID-19 pandemic in Singapore. Due to the stressors inflicted by the additional workload, participants felt that the program was delivered at an opportunistic time to support their psychological well-being:

I think you kind of met me at the right time and I feel that I need to self-improve.

Participant 3, male, Chinese, nurse

In particular, participants appreciated how the contents were relatable to their concerns and felt that they were able to translate their newly acquired theoretical knowledge to an actual situation:

I really appreciate the teamwork and emotional regulation, like the ones I could really practice, putting time for myself, things like that.

Participant 6, male, Chinese, nurse

### Theme 3: Promoting Participants’ Workforce Readiness

#### Overview

The final theme describes how the BRAW program has influenced participants’ readiness to maintain in the workforce. Through participants’ narratives, the BRAW program has a profound impact on their resilience, intent to stay, and employability. This theme is further elaborated in 3 subthemes, namely, drawing resilience in times of adversity, promoting intent to stay in health care, and becoming future-ready.

#### Drawing Resilience in Times of Adversity

The BRAW program instilled numerous positive aspects in participants. As participants translated their newly acquired knowledge into practice, they demonstrated resiliency by overcoming the challenges and difficulties experienced in the workplace:

Yup, especially when dealing with negative emotions and how to bounce back up again.

Participant 1, male, Chinese, nurse

When asked about the extent of the improvements, the majority of the participants felt noticeable improvements. For instance, they observed an evident increase in their ability to overcome situations:

In the past...I take quite a while to recover...Then, nowadays, it’s a bit better, even though I think about it, I can move on from it. And I can have a more positive mindset about it. So, I don’t blame myself for something that happened, or I don’t dwell on the thing that happened. Instead, I focused on the future, like if it happens again, what can I do.

Participant 13, female, Chinese, audiologist

#### Promoting Intent to Stay in Health Care

Participants also felt that the BRAW program supported their resilience to remain steadfast in the health care sector. This was an interesting viewpoint expressed by most participants because it proposes that the improvement of psychological well-being has increased their intent to stay in their current role:

This course [referring to the BRAW program] actually helps me dispel away negative thoughts, put things in perspective, and reframe my mind away so that I can still go through the job.

Participant 14, female, Malay, medical technician

However, most of the participants also felt that resilience training alone may not be sufficient to influence their intent to stay. Instead, one’s intent to stay may be influenced by a larger environmental factor such as management-related reasons:

The management did not do anything, so I feel that I should just quit this organization because they don’t take care of us.

Participant 25, male, Malay, nurse

#### Becoming Future-Ready

The majority of the participants felt that resilience is a form of a positive attribute. When asked if being resilient is an important factor in securing employment, participants felt that resiliency was a personal competency and may have indirect impacts on getting one employed:

I won’t say, it’s directly, okay, this [referring to the BRAW program] will help you get the job, but it’s more of like okay, it helps you work on yourself as a person. So, that indirectly translates to being a more employable person.

Participant 13, female, Chinese, audiologist

Nevertheless, participants perceived that the contents of the BRAW program could help shape an individual’s emotional quotient. This may translate to the development of one’s leadership skills:

It [referring to the BRAW program] shapes a person who has a lot of EQ and understanding...So, I think it does make, if you can master these techniques very well, I do believe that it can make you a better leader.

Participant 12, male, Chinese, respiratory therapist

## Discussion

### Principal Findings

This qualitative study aimed to explore HCPs’ experiences of participating in the web-based BRAW program during the COVID-19 pandemic. Based on the framework analysis, participants alluded to the importance of the various web-based elements that supported their internalization of the resilience processes. Particularly, the asynchronous and self-paced nature and applicable materials supported participants’ continued engagement with the BRAW program. Finally, after attending the BRAW program, participants became resilient, had greater intent to stay, and were future-ready.

With regard to the web-based elements, the availability of different web-based learning tools has supported participants’ learning. This finding was consistent with prior research that evaluated web-based resilience programs [22,40]. Several key characteristics of web-based learning stood out. First, participants alluded to the importance of short attention-requiring materials such as videos, which was similarly reported in other studies [40,41]. Second, participants credited the availability of quizzes and homework that supplemented their learning. Homework and quizzes can augment the learning process by allowing individuals to apply their newly acquired knowledge [42,43]. Despite the benefits, several participants were overwhelmed by the number of tasks (eg, videos, quizzes, homework, and forum). A unique finding from this study was regarding the nomenclature of the tasks. Particularly, participants mentioned that the term “homework” can be considered a chore and may not be preferred in this form of program. This could be due to participants’ experiences with homework during their schooling years, where numerous negative emotions were associated with that term [44,45].

With regard to the contents, participants credited how the relatability and applicability of the BRAW contents were facilitators for completion. This is an important aspect, as several studies have echoed the importance of providing contextually relevant materials for participants [41,46], and this will facilitate participants’ understanding and transferability of their newly acquired skills. Furthermore, participants appreciated the resilience strategies and applied them in the workplace. For example, the provision of easily replicable strategies such as the application of the problem-solving algorithm was helpful for the participants [27,47].

With regard to the features, the web-based BRAW program was designed as asynchronous and self-paced training for several reasons, such as wider outreach and the presence of the COVID-19 pandemic. The use of a web-based approach was verbalized as an enabler for HCPs to complete the program, which was consistent with other studies [22,48]. In addition, a web-based approach provided HCPs with an opportunity to learn during the COVID-19 pandemic when induced social distancing measures were required. More importantly, the nature of the BRAW program promoted participants’ autonomy and allowed them to gain control over their schedules. This could stimulate personalized learning, which resulted in positive effects on one’s learning outcomes [49,50]. However, despite this, most of the participants also experienced conflicting priorities and were unable to timely participate in the web-based BRAW program. Considering that participation in programs of such nature is of lower priority than their formal work-related commitments, this may have led to their reduced participation [22,27].

Through participants’ narratives, this study also unveiled the positive effects of the web-based BRAW program on their resilience, intent to stay, and employability. From a resilience perspective, the program provided participants with skills ranging from personal (eg, cognitive restructuring), relational (eg, teamwork), and environmental (eg, workplace environment) that promoted their resilience. Based on the resilience theory [11], the introduction of such resilience protective factors can promote resilience. Interestingly, participants’ resilience could also be influenced by the recognition of their resilient potential. Several studies have suggested how the introduction of resilience programs has led to participants becoming aware of their internal strengths and how this influences their resilience [22,51].

Moreover, the web-based BRAW program introduced techniques to enhance cognitive restructuring, positivity, and happiness, and this could be a plausible explanation for improving participants’ intent to stay. Despite the dynamic and stressful health care environment, these techniques potentially supported participants’ positive reframing of a seemingly negative situation [15,31]. Furthermore, it can have positive direct or mediating effects on one’s intent to stay by improving one’s optimism and positivity [52,53]. However, participants also surfaced that macro-organization factors such as hospital administration are factors that may negatively affect their intent to stay [54,55]. While not directly explored in other qualitative evaluations of resilience programs, this study found that the web-based BRAW program has encouraging effects on participants’ employability and future readiness. This could be attributed to the introduction of various noncognitive skills such as problem-solving and emotion regulation. More literature has highlighted the pivotal role of noncognitive skills on employment outcomes [56,57].

Based on the findings from this qualitative study, several implications for future resilience programs are outlined. First, HCPs continue to experience mental exhaustion and distress due to the immense workload caused by the COVID-19 waves, and the delivery of a web-based program targeting mental well-being is practical and should be implemented. Second, from a feature perspective, an asynchronous and self-paced program is an acceptable and feasible approach. However, to reduce any potential conflicting work commitments, participants should be provided with protected time to complete these programs. Third, web-based learning should be supplemented by various engagement tools, and it will be helpful to redesignate homework as self-help exercises or tasks to reduce the negative connotation associated with homework. Next, from a content perspective, contextualized personal, relational, and environmental resilience materials should be introduced. Thus, conducting a needs analysis would be necessary to ensure that the resilience program remains acceptable to the target population. In addition, there should be an introduction of technical terms for participants who may not be familiar with the materials. Finally, as resilience programs focus on building an individual’s strengths, it will be important that health care administrators consider building supportive workplace environments to complement resilience programs.

### Limitations

This study has several limitations, and results need to be interpreted with caution. First, this qualitative study explored participants’ experiences of 1 web-based resilience program, and its findings may not be transferable to other settings. Despite this, our findings may provide insight on the design of future psychosocial web-based interventions. Second, most of them were female and ethnic Chinese participants, thereby resulting in an underrepresentation of other sex and ethnic groups. Nevertheless, a rigorous purposive sampling approach was undertaken to ensure that there is a good representation of individuals across various age groups and professions. Finally, this study was limited to a 1-time point and may not be able to encapsulate the long-term effects of the BRAW program on the participants.

### Conclusions

This study presented a qualitative evaluation of a web-based BRAW program using framework analysis. Although there were several highlighted facilitators and barriers, the findings show that an asynchronous, self-paced resilience program can be a useful tool in supporting the well-being of HCPs during the COVID-19 pandemic. However, it will be important to ensure that contextually relevant materials, supported by other appropriate web-based engagement tools, such as quizzes and practical exercises are provided to promote learning in a web-based environment. Further work is needed to explore how macro-organization factors can be embedded in resilience programs to promote HCPs’ resilience and well-being.

## Appendix

Multimedia Appendix 1 COREQ (Consolidated Criteria for Reporting Qualitative Research) checklist.

Multimedia Appendix 2 Table S1. Coding tree.

## Abbreviations

BRAW: Building Resilience At Work
COREQ: Consolidated Criteria for Reporting Qualitative Research
HCP: health care professional
