# Supplementary material for: Unpacking the Experiences of Health Care Professionals About the Web-Based Building Resilience At Work Program During the COVID-19 Pandemic: Framework Analysis
Source: JMIR Med Educ. 2024 Jan 31;10:e49551. doi: 10.2196/49551 (PMC10867752; doi:10.2196/49551)
Supplement: Multimedia Appendix 2 [file mededu_v10i1e49551_app2.docx]

**Table S1.** Coding tree.

| Process evaluation framework: themes and subthemes | | | Verbatim quotations |
| --- | --- | --- | --- |
| **Contextual factors and implementation** | | | |
|  | **Learning from web-based tools** | | |
|  |  | Engaging with web materials | - “The use of graphics was quite good, the animations and all, so like, it kept me wanting to finish watching, not like stop halfway. Yeah...the pace was also good, and like, just nice, not too much information overload” [Participant 24, female, Chinese, nurse]. - “Because ‘homework’ it sounds like ‘tsk,’ erm, like a chore to be done, you know, but ‘reflection’ is like, you reflect on what you-you-you need to do. So, sounds more forgiving” [Participant 26, female, Malay, nurse]. |
|  |  | Internalizing the resilience process | - “Just by plain reading the question, it may set you thinking, you see. You don’t know what’s happening or your subconscious, you’re already motivated right, you learn some new content. And that homework may actually be building synapses, you know, trying at the backend that you don’t know about” [Participant 10, male, Malay, physician]. - “When it gets a little bit more ‘science-y,’ like the brain and then they tell you, I don’t know all the words, I don’t remember, but like the brain and then, certain kinds of thoughts and all that. Then, those kinds of stuff, no, like I haven’t heard of that before” [Participant 15, female, Indian, clinical researcher]. - “Especially some of the terms, erm, maybe a bit technical? I’m not that acquainted. So, it [referring to the quizzes] allows me to clarify, review and understand and get it correct” [Participant 8, female, Chinese, clinical administrator]. |
| **Mechanism of impact and contextual factors** | | | |
|  | **Interacting with the BRAW program** | | |
|  |  | Appreciating the asynchronous self-paced program | - “Healthcare workers are busy, so they don’t have to find a specific day and time to attend an intervention, whether be it online or on-site, face-to-face or whatever, so having something that you can access on your own time and target is good” [Participant 4, female, Chinese, clinical researcher]. - “We are really packed and rushed at work, and there’s a lot of multitasking. It’s like very draining at work. I think the shifts also, so you do rotating shifts. So, it’s quite tiring after work to find time.” [Participant 5, female, Chinese, nurse] - “These sessions were to be interactive whereby we can do it via Zoom, to share every participant’s experience, it would be even better” [Participant 28, female, Chinese, nurse]. |
|  |  | Relating to the applicability of the contents | - “I think you kind of met me at the right time and I feel that I need to self-improve” [Participant 3, male, Chinese, nurse]. - “I really appreciate the teamwork and emotional regulation, like the ones I could really practice, putting time for myself, things like that” [Participant 6, male, Chinese, nurse]. |
| **Outcome** | | | |
|  | **Promoting participants’ workforce readiness** | | |
|  |  | Drawing resilience in times of adversity | - “Yup, especially when dealing with negative emotions and how to bounce back up again” [Participant 1, male, Chinese, nurse]. - “In the past...I take quite a while to recover...Then, nowadays, it’s a bit better, even though I think about it, I can move on from it. And I can have a more positive mindset about it. So, I don’t blame myself for something that happened, or I don’t dwell on the thing that happened. Instead, I focused on the future, like if it happens again, what can I do” [Participant 13, female, Chinese, audiologist]. |
|  |  | Promoting intent to stay in health care | - “This course [referring to the BRAW program] actually helps me dispel away negative thoughts, put things in perspective, and reframe my mind away so that I can still go through the job” [Participant 14, female, Malay, medical technician]. - “The management did not do anything, so I feel that I should just quit this organization because they don’t take care of us” [Participant 25, male, Malay, nurse]. |
|  |  | Becoming future-ready | - “I won’t say, it’s directly, okay, this [referring to the BRAW program] will help you get the job, but it’s more of like okay, it helps you work on yourself as a person. So, that indirectly translates to being a more employable person” [Participant 13, female, Chinese, audiologist]. - “It [referring to the BRAW program] shapes a person who has a lot of EQ and understanding...So, I think it does make, if you can master these techniques very well, I do believe that it can make you a better leader” [Participant 12, male, Chinese, respiratory therapist]. |

^a^BRAW: Building Resilience At Work
